# Comparison of immunohistochemistry and Ziehl‐Neelsen staining for detecting the distribution of *Mycobacterium avium* subsp *avium* in naturally infected domestic Pekin ducks (*Anas platyrhynchos domestica*)

**DOI:** 10.1002/vms3.223

**Published:** 2019-11-26

**Authors:** Dekang Zhu, Hongxi Chen, Xumin Ou, Mafeng Liu, Mingshu Wang, Xinxin Zhao, Renyong Jia, Shun Chen, Kunfeng Sun, Qiao Yang, Ying Wu, Xiaoyue Chen, Anchun Cheng

**Affiliations:** ^1^ Research Center of Avian Diseases College of Veterinary Medicine Sichuan Agricultural University Chengdu Sichuan China; ^2^ Key Laboratory of Animal Disease and Human Health of Sichuan Province Chengdu Sichuan China; ^3^ Institute of Preventive Veterinary Medicine Sichuan Agricultural University Chengdu Sichuan China

**Keywords:** distribution, domestic ducks, haematoxylin and eosin, immunohistochemistry, *Mycobacterium avium* subsp *avium*, Ziehl‐Neelsen staining

## Abstract

In order to detect the distribution of *Mycobacterium avium subsp avium* (MAA) in naturally infected domestic Pekin ducks, immunohistochemistry (IHC) and Ziehl‐Neelsen (ZN) staining were used and compared. Six organs, the liver, spleen, lung, kidney, duodenum and pectoralis muscle, were collected from naturally infected Pekin ducks. Paraffin embedded tissues were examined, and the results were compared. Statistical analysis was performed using Chi‐Square test. The results showed that the detection rates by IHC were similar with ZN staining in liver, lung, spleen and pectoralis muscle, but the detection rates by IHC were much higher than ZN staining in kidney and duodenum (*p* = .013, *p* = .0044). The liver (87.5%) and lung (81.3%) had the highest detection rates. Acid‐fast bacilli (AFB) were primarily found intracellularly in six organs using ZN staining. Similarly, the MAA antigens in those selected organs were also detected in the cytoplasm with different cell types. Specifically, MAA antigen was distributed in epithelioid macrophages and necrotic centres within the liver, lung, spleen and kidney, while they were observed in macrophages of the lamina propria and duodenal glands and degenerative myocytes in the pectoralis muscle. This comparative study provides an important insight into the distribution of MAA in infected domestic ducks and indicates that the detection rate by IHC was higher than that of ZN staining.

## Graphical abstract

This comparative study provides an important insight into the distribution of MAA in infected domestic ducks and indicate that the detection rate by IHC was higher than that of ZN staining.

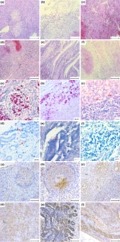

## 1 INTRODUCTION

Avian tuberculosis is a contagious disease caused by *Mycobacterium avium* subsp *avium* (MAA). The primary modes of transmission are via oral and airborne routes. Avian tuberculosis can result in progressive weight loss, reduction in egg production, and ultimately, mortality of birds. It has been reported that avian tuberculosis can infect domestic poultry (Tell, Woods, & Cromie, 2001), companion psittacine birds (Lennox, 2007) and captive exotic birds (Montali, Bush, Thoen, & Smith, 1976). However, disease susceptibility varies from species to species. While it has been reported that domestic geese and ducks are moderately resistant to *M.* *avium* (Hejlícek & Treml, 1995), an outbreak of avian tuberculosis in a commercial domestic duck flock was recently reported (Song et al., 2016; Zhu et al., 2016). A strain has been isolated from organs of infected ducks and sequenced using PacBio single‐molecule real‐time sequencing technology. Based on the complete genome of isolates (GenBank accession no. CP016396), it has been determined that the subspecies identified is MAA (Song et al., 2016).

To the authors’ knowledge, detection of MAA in ducks using immunohistochemistry (IHC) has not been reported. However, exploring MAA distribution in the organs of naturally infected domestic ducks is necessary to understand avian tuberculosis. Therefore, Ziehl‐Neelsen (ZN) staining and IHC staining of a variety of organs were compared for detecting MAA.

## 2 MATERIALS AND METHODS

Sixteen MAA‐infected Pekin breeder ducks from a duck farm in Sichuan Province of China and two non‐infected ducks from another flock of the same age were used for the study. All 18 ducks were anesthetized (pentobarbital sodium, 30 mg/kg, administered intravenously) and humanely killed by cervical dislocation. Liver, spleen, lung, kidney, duodenum and pectoralis muscle were collected at the same regions from all ducks. These organs were fixed in 4% paraformaldehyde within 24 hr followed by standard paraffin embedding. Then, sections of 5 μm were cut and were stained with Haematoxylin and Eosin (H&E), ZN and for IHC assays.

Two healthy male New Zealand White rabbits (2.5 kg, obtained from Chengdu Dashuo Experimental Animal Co Ltd.) were used to prepare rabbit anti‐MAA IgG. Rabbits were injected via the ear with *M.* *avium* strain, which was inactivated by 0.4% formaldehyde, every three days for a total of 21 days. The doses for the seven injections of *M.* *avium* were as follows: 1 × 10^7^ CFU, 2 × 10^7^ CFU, 5 × 10^7^ CFU, 1 × 10^8^ CFU, 1.5 × 10^8^ CFU, 2 × 10^8^ CFU and 3 × 10^8^ CFU, respectively. The rabbits were anesthetized with an intravenous injection of sodium pentobarbital (25 mg/kg), and blood was collected from the carotid artery at 24 days. Serum was collected by centrifugation at 1,000*g* for 5 min at 4°C The rabbit anti‐MAA IgG was purified from antiserum using a protein A sepharose FF^®^ adsorption column.

IHC staining of the infected organs was conducted as reported previously (Chen et al., 2009). Briefly, 5 μm paraffin sections were deparaffinized in xylene and rehydrated in graded alcohol. For antigen retrieval, sections were boiled in 10 mmol citrate buffer (pH 6.0) for 15 min. Endogenous peroxidase was then blocked with 3% H_2_O_2_ in methanol for 20 min at room temperature. Sections were then incubated in 10% bovine serum albumin (BSA) (Boster) blocking solution at 37°C for 30 min. Sections were then incubated with the rabbit anti‐MAA IgG (1:10 dilution) overnight at 4°C. Sections were then incubated with goat anti‐rabbit IgG (1:500 dilution) conjugated with hyperoxide peroxidase (HRP) (Boster, Wuhan, China) for 1 hr at 37°C. The sections were then visualized with diaminobenzidine (Boster) for 4 min and counterstained with haematoxylin. The tissues of two non‐infected ducks were also stained following the above steps in order to act as negative controls. All infected tissues were also incubated with phosphate buffered saline (PBS), which replaced the primary antibody to act as secondary control.

All sections were examined using an optical microscope (Nikon Eclipse 80i, Japan) at ×400 (H&E and IHC) or ×1,000 (ZN) magnification, and images were obtained using a SPOT Flex Camera (Diagnostic Instruments). The results of IHC were semi‐quantified using an H‐score reported independently by two pathologists (Bwala, Clift, Duncan, Bisschop, & Oludayo, 2012; Shousha, 2008). The H‐score system was based on the proportion and intensity of brown staining cells. The intensity of the stain was graded as: 0 = negative, 1 = weak intensity, 2 = intermediate intensity and 3 = strong intensity. H‐score = (% of cells stained at intensity 1 × 1) + (% of cells stained at intensity 2 × 2) + (% of cells stained at intensity 3 × 3). A H‐score of <10 was considered negative (−), a score of 10–100 as weakly positive (1+), a score of 101–200 as moderately positive (2+) and a score of 201–300 as strongly positive (3+).

Statistical analysis was performed using IBM SPSS Statistics for Windows, Version 24.0 (IBM Corp.). The Chi‐Square test was used to determine the significant differences between IHC and ZN detection methods. *p* < .05 was considered statistically significant.

## 3 RESULTS

**Figure 1:**
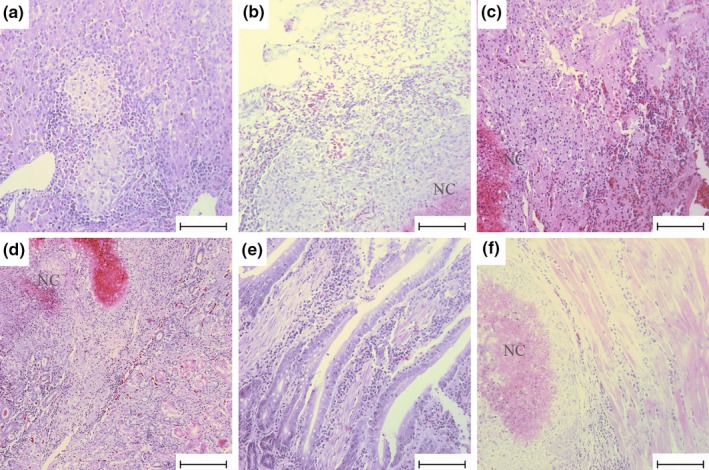
Haematoxylin and eosin (H&E) staining of different organs in domestic Pekin ducks infected with MAA. (a) Granulomas without necrosis in liver. (b) Granulomas with necrosis in lung. (c) Granulomas with necrosis in spleen. (d) Granulomas with necrosis in kidney. (e) No granulomas in duodenum. (f) Granulomas with necrosis in pectoralis muscle. Bar = 75 μm, magnification ×400. NC, Necrotic Center

Histopathological results revealed granulomas without necrosis (Figure 1a) and granulomas with necrosis (Figure 1b–d and f) in the liver, lung, spleen, kidney and pectoralis muscle. Interestingly, no granulomas were observed in the duodenum on microscopic examination (Figure 1e). Granulomas with necrosis were characterized by caseous necrosis that contained nuclear debris, surrounded by epithelioid macrophages and lymphocytes. Many small necrotizing foci appeared to fuse, forming large necrotizing granulomas, as observed in the spleen (Figure 1c), kidneys (Figure 1d) and pectoralis muscle (Figure 1f). The pectoralis muscle, with tubercle nodules, showed caseous necrosis that was surrounded by degenerative myocytes, with some lymphocytic infiltration, rather than the presence of granulomas (Figure 1f). Specifically, multinucleated giant cells were rarely observed in granulomas of the liver, lung, spleen and kidney.

**Figure 2:**
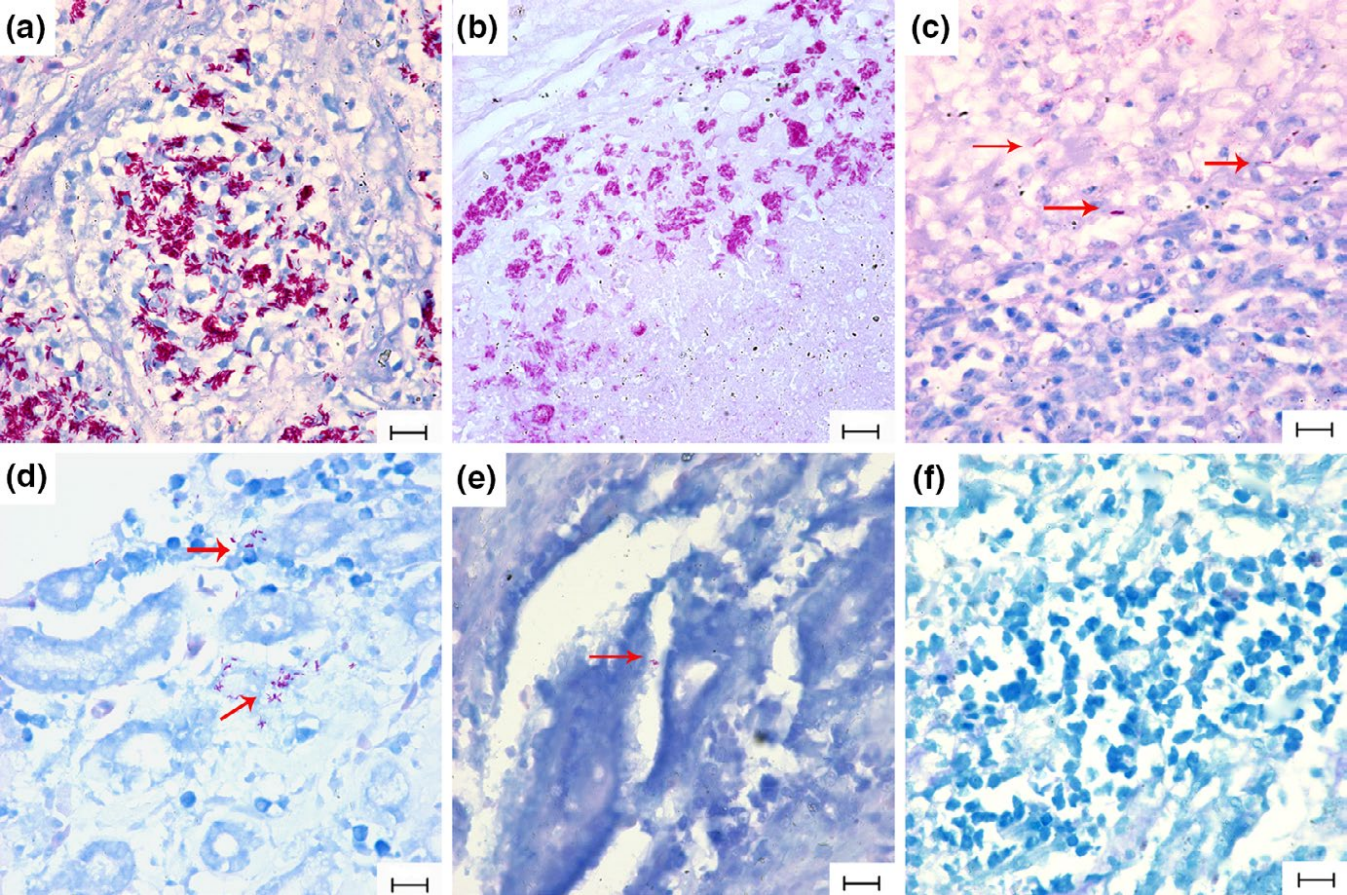
Ziehl‐Neelsen (ZN) staining of different organs of domestic Pekin ducks infected with MAA. Representative images of liver (a), lung (b), spleen (c), kidney (d), duodenum (e) and normal liver (f). Bar = 30 μm, magnification ×1,000. Red arrows indicate the AFB

ZN staining showed that acid‐fast bacilli (AFB) were present in all six organs examined. AFB were primarily found intracellularly, with few AFB found extracellularly. However, the number of AFB in each organ was different. Large concentrations of AFB were observed in the liver (Figure 2a), lung (Figure 2b) and pectoralis muscle, while, few were found in the spleen (Figure 2c), kidneys (Figure 2d) and duodenum (Figure 2e). The detection rates were different among organs. For example, 87.5% (14/16) of livers demonstrated the presence of AFB, while only 18.8% (3/16) of duodenum had detectable AFB (Table 1).

**Table 1:** Percentage of positively stained organs of the MAA infected domestic Pekin ducks by ZN staining and IHC

	Total	ZN		IHC		*χ* ^2^	*p*
		Positive	Percentage	Positive	Percentage		
Liver	16	14	87.5%	14	87.5%	NA	NA
Lung	16	12	75.0%	13	81.3%	0.18	.69
Spleen	16	8	50.0%	11	68.8%	1.17	.28
Kidney	16	5	31.3%	12	75.0%	6.15	.013
Duodenum	16	3	18.8%	11	68.8%	8.13	.0044
Pectoralis muscle	16	12	75.0%	12	75.0%	NA	NA

Note

Percentage: the ratio of the number of tissues had positive staining to the total number of tissues. The Chi‐Square test: *p* < .05 is significance. NA, not applicable.

**Figure 3:**
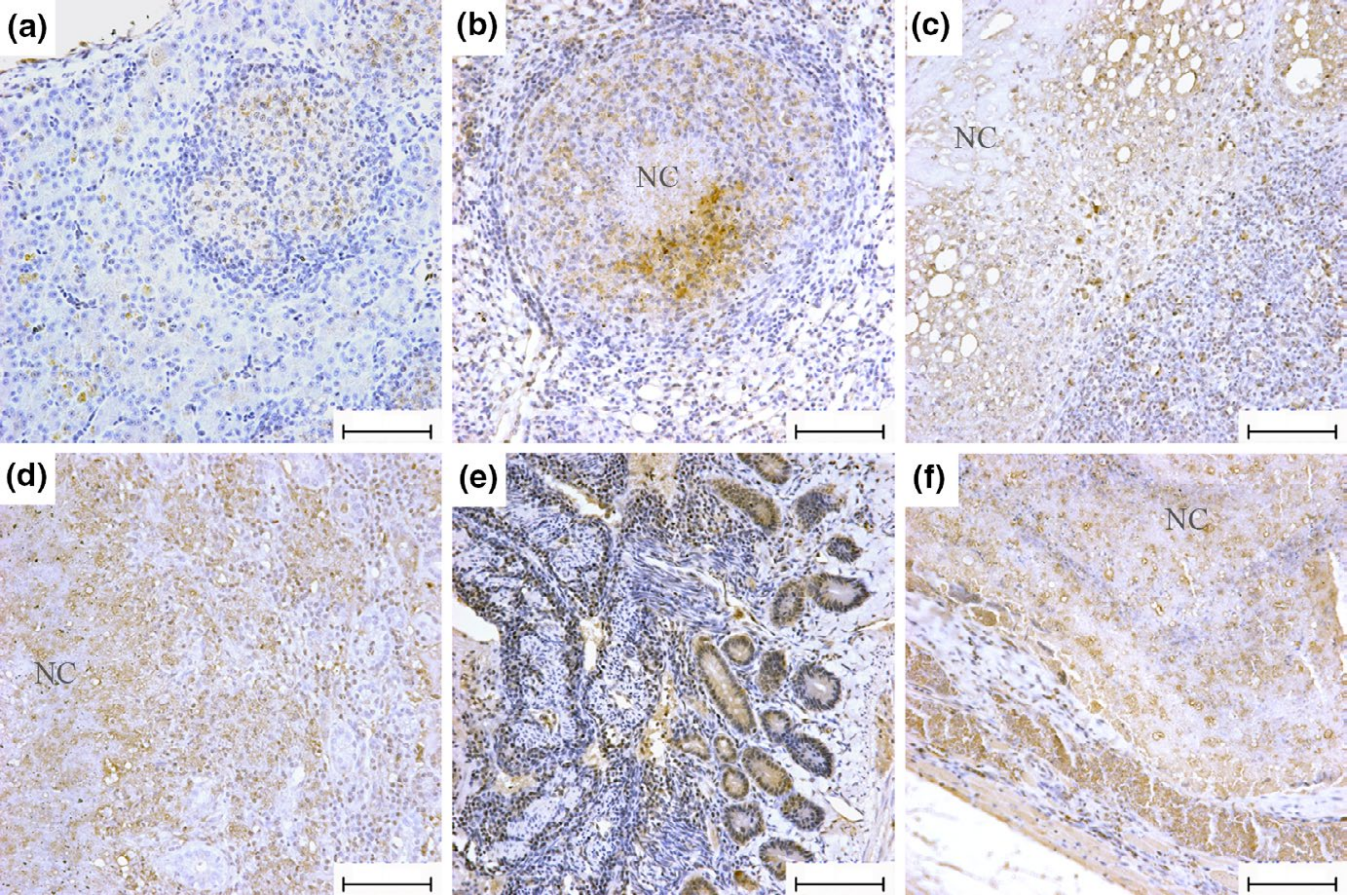
IHC staining of different organs of domestic Pekin ducks infected with MAA. (a) liver; (b) lung; (c) spleen; (d) kidney; (e) duodenum; (f) pectoralis muscle. Bar = 75 μm, magnification ×400. NC, Necrotic Centre

MAA infection of Pekin ducks was further examined by IHC. Similar to ZN staining, positive staining was observed in all six organs examined (Figure 3). All negative controls showed no brown staining. In liver sections with non‐necrotizing granulomas, positive staining was primarily observed in the cytoplasm of epithelioid macrophages and denatured hepatocytes (Figure 3a). In lung (Figure 3b), spleen (Figure 3c) and kidney sections (Figure 3d) with necrotizing granulomas, positive staining was detected in the cytoplasm of epithelioid macrophages as well as in necrotic centres. Positive staining was also observed in macrophages of the lamina propria and duodenal glands (Figure 3e), as well as in degenerative myocytes in the pectoralis muscle (Figure 3f). Consistent with ZN staining, immunostaining showed that the less abundant stainings were observed in the liver, lung and pectoralis muscle, while relatively slight brown stainings were observed in the spleen, kidney and duodenum (Table 2). However, the MAA‐positive detection rates among the six organs showed no difference (*p* > .05).

**Table 2:** IHC analysis of positively stained samples of liver, spleen, lung, kidney, duodenum and pectoralis muscle in the MAA‐infected domestic Pekin ducks

	Total	Number (percentage) of positively labelled organs				
		－	1+	2+	3+	Positive
Liver	16	2 (12.5%)	4 (25.0%)	4 (25.0%)	6 (37.5%)	14 (87.5%)
Lung	16	3 (18.8%)	4 (25.0%)	4 (25.0%)	5 (31.3%)	13 (81.3%)
Spleen	16	5 (31.3%)	5 (31.3%)	3 (18.8%)	3 (18.8%)	11 (68.8%)
Kidney	16	4 (25.0%)	5 (31.3%)	6 (37.5%)	1 (6.3%)	12 (75.0%)
Duodenum	16	5 (31.3%)	3 (18.8%)	4 (25.0%)	4 (25.0%)	11 (68.8%)
Pectoralis muscle	16	4 (25.0%)	2 (12.5%)	5 (31.3%)	5 (31.3%)	12 (75.0%)

Note

“−”: negative, a score of <10; “1+”, weakly positive, a score of 10–100; “2+”, moderately positive, a score of 101–200; “3+”, strongly positive, a score of 201–300.

To measure the difference in detection rates, a comparative study between IHC and ZN was conducted. As shown in Table 1, only in the liver and pectoralis muscle, where large concentrations of AFB were identified, were the detection rates of IHC and ZN staining identical. While in lung and spleen where found moderate concentrations of AFB, the detection rates of IHC (81.3% and 68.8%) were slightly higher than those of ZN staining (75.0%; *p *= .69 and 50.0%; *p *= .28, respectively). In kidney and duodenum where found few AFB, the detection rates of IHC (75.0% and 68.8%) were observably higher than those of ZN staining (31.3%; *p* = .013 and 18.8%; *p *= .0044, respectively).

## 4 DISCUSSION

In general, multinucleated giant cells are commonly found in birds with avian tuberculosis. Multinucleated giant cells play an important role in resisting tuberculosis because they may limit the growth and spread of *Mycobacterium tuberculosis* (Dannenberg, 2006). Indeed, the presence of multinucleated giant cells has been reported in wild ducks infected with *Mycobacterium avium* (Roffe, 1989). However, multinucleated giant cells were rarely observed in tissues of naturally infected Pekin ducks in this study, which is similar to a previous study (Saggese et al., 2007). We proposed a possible explanation that the stimulus that induces multinucleated giant cell formation is reduced. It is therefore possible that a defect in multinucleated giant cell formation in Pekin ducks increased their susceptibility to MAA infection, although it has been reported that geese and ducks are moderately resistant to MAA (Hejlícek & Treml, 1995). Some reports also claimed that decreased genetic diversity of commercial ducks leads to an increased susceptibility to MAA infection (Acevedo‐Whitehouse, Gulland, Greig, & Amos, 2003; Bonneaud, Pérez‐Tris, Federici, Chastel, & Sorci, 2006; Miller & Lambert, 2004). In addition, we suspected that this isolated strain is very virulent, but further studies are needed to confirm this suspicion.

The detection rates of MAA in spleen and duodenum were lower in both methods. The reason may be that there are a large number of lymphocytes and macrophages in the tissue structure of the spleen and duodenum, which is more effective in inhibiting the colonization of bacteria in tissues.

In this study IHC detected more MAA than ZN staining in the kidney and duodenum. It may suggest that immunostaining may be more sensitive than ZN staining in detecting MAA infection. As ZN staining requires bacteria with intact cell walls, which depend on the formation of mycolic acid‐carbol fuchsin complex, this may lead to a comparatively lower detection rate (Martinson et al., 2008). Indeed, mycobacteria tend to lose acid‐fast ability under stressful environments (Deb et al., 2009). In contrast, IHC can detect all types of bacterial proteins without needing intact cell walls and therefore provides a higher sensitivity, which is also in accordance with previous reports (Thoresen, Falk, & Evensen, 1994). Considering the technical advances in detecting MAA and relative higher detection rates, IHC will provide the potential to detect low concentrations of MAA, especially at early stage of infection.

The ducks in this study were naturally infected and as such this study did not investigate the dynamic distribution of MAA in ducks. An artificial infection model for avian tuberculosis will be established in the future. In addition, more bioinformatics analysis and identification of virulence of MAA are of significance to the understanding of the bacteria.

## 5 CONCLUSION

The IHC and ZN staining methods can be used to detect the distribution of MAA in tissues of the naturally infected domestic Pekin ducks and the MAA antigens were primarily detected in the epithelioid macrophages in selected organs. There was no difference in MAA‐positive detection rates between the six organs (liver, spleen, lung, kidney, duodenum and pectoralis muscle). Additionally, this study confirms that the detection rate of MAA in naturally infected domestic ducks by IHC was higher than that of ZN staining. These findings lay the foundation for further research on the pathogenesis of Avian tuberculosis.

## CONFLICT OF INTEREST

The authors declare that they have no competing interests.

## ETHICAL STATEMENT

The animal‐use procedures were approved by the Animal Ethics Committee of the Sichuan Agricultural University.
