# Supplementary material for: Comparison of immunohistochemistry and Ziehl‐Neelsen staining for detecting the distribution of Mycobacterium avium subsp avium in naturally infected domestic Pekin ducks (Anas platyrhynchos domestica)
Source: Vet Med Sci. 2019 Nov 26;6(2):242–7. doi: 10.1002/vms3.223 (PMC7196683; doi:10.1002/vms3.223)
Supplement: Supplementary file 1 [file VMS3-6-242-s001.docx]

**Supplementary Table 1.** Comparison of detection rates among the six organs of the MAA infected domestic Pekin ducks by IHC

|  | *P* value | | | | | |
| --- | --- | --- | --- | --- | --- | --- |
| Organs | Liver | Lung | Spleen | Kidney | Duodenum | Pectoralis muscle |
| Liver | - | 0.63 | 0.20 | 0.37 | 0.20 | 0.37 |
| Lung | 0.63 | - | 0.41 | 0.67 | 0.41 | 0.67 |
| Spleen | 0.20 | 0.41 | - | 0.69 | NA | 0.27 |
| Kidney | 0.37 | 0.67 | 0.69 | - | 0.69 | NA |
| Duodenum | 0.20 | 0.41 | NA | 0.69 | - | 0.69 |
| Pectoralis muscle | 0.37 | 0.67 | 0.27 | NA | 0.69 | - |

NOTE: The Chi-Square test: *P* < 0.05 is significance. NA: not applicable.
